# One-Dimensional Iodoantimonate(III) and Iodobismuthate(III) Supramolecular Hybrids with Diiodine: Structural Features, Stability and Optical Properties

**DOI:** 10.3390/molecules27238487

**Published:** 2022-12-02

**Authors:** Nikita A. Korobeynikov, Andrey N. Usoltsev, Pavel A. Abramov, Maxim N. Sokolov, Sergey A. Adonin

**Affiliations:** Nikolaev Institute of Inorganic Chemistry SB RAS, Novosibirsk 630090, Russia

**Keywords:** antimony, bismuth, polynuclear complexes, halide complexes, non-covalent interactions

## Abstract

Two isostructural pairs of supramolecular iodoantimonate(III) and iodobismuthate(III) complexes with I_2_ units “trapped” in solid state via halogen bonding—Cat_3_[[M_2_I_9_](I_2_)} (Cat = tetramethylammonium and 1-methylpyridinium, M = Sb(III) and Bi(III)) were prepared. For all compounds, values of optical band gaps were determined, together with thermal stability; the complexes were additionally characterized by Raman spectroscopy.

## 1. Introduction

Within the past decades, there has been an ongoing growth of interest on halide complexes of p-block elements. The motivation of research in this field is mostly explained by the fact that iodometalates (in particular, iodoplumbates(II) [1,2,3,4]) can be used as components of solar cells and/or photodetectors—so-called perovskite photovoltaics constituted an area that has developed extremely rapidly [5,6,7,8,9,10,11,12].

One of the problems in this area is the fact that “true” isotropic 3D halometalates (with all covalent bonds), ideally suitable for the use as light absorbers in photovoltaic devices, are extremely rare: those are formed by either Sn(II) or Pb(II) with a very limited number of small organic (such as methylammonium [13,14]) or inorganic (such as cesium [3,15]) cations. This fact encourages the search for alternative candidates in this role. Halometalates of Sb, Bi and other p-elements have been extensively tested [16], mostly revealing low-to-moderate efficiencies. This is generally explained by the less favorable molecular structure of these compounds (those are 0D, 1D or, rarely, 2D).

There are different approaches towards the overcoming of this obstacle. Recently, Shevelkov et al., presented the first supramolecular iodobismuthate(III) complexes containing diiodine units [17,18,19]. In such hybrids, I_2_ is “captured” in solid state, forming halogen bonding with iodometalate anions and acting therefore as “linker”. Such structures feature therefore higher dimensionality of halometalate framework which is, as mentioned above, desirable for the enhancement of photovoltaic properties. It is also notable that these hybrids usually feature lower optical band gaps than “pure iodobismuthates” (<1.7 eV) and, at the same time, can be thermally stable enough to be used in solar cells.

Performing research in the field of polyhalogen-halometalates since the last years, we confirmed that representatives of this class indeed can be suitable for photovoltaic applications (mostly as photodetectors) [20,21]. Our work with polyiodo-iosobismuthates(III) resulted in the discovery of new structural types, including 1D and 2D supramolecular frameworks [22]. However, the number of these compounds is yet small. Moreover, those are virtually unknown for the closest neighbor of Bi(III)—antimony, which also readily forms halide complexes. On this reason, we decided to try preparing the pairs of polyiodo-iodometalates of Sb(III) and Bi(III) in order to compare their structural features (suggesting that those can be isostructural), as well as physical properties.

Hereby, we present four new supramolecular halometalates: (Me_4_N)_3_{[M_2_I_9_](I_2_)} (M = Sb (**1**), Bi (**2**)) and (1-MePy)_3_{[M_2_I_9_](I_2_)} (M = Sb (**3**), Bi (**4**)). Those were characterized by X-ray diffractometry, Raman and diffuse reflectance spectroscopy, as well as thermogravimetric analysis (TGA).

## 2. Experimental Part

The chemicals were purchased from commercial sources and used without additional purification. Solvents were purified according to the standard procedures. The 1-methylpyridinium iodide was prepared by reaction of pyridine and methyl iodide (1:1.05) following the standard method of alkylation of substituted pyridines. In all cases, concentrate HI was used.

### 2.1. Synthesis of ***1***

We dissolved 58 mg (0.2 mmol) of Sb_2_O_3_, 121 mg (0.3 mmol) of Me_4_NI and 51 mg (0.2 mmol) of I_2_ in 4.5 mL of HI (70 °C, stirring, 30 min). Resulting solution was slowly cooled to r.t., resulting in formation of dark crystals within 1 d. Yield 79%. For C_12_H_36_N_3_Sb_2_I_11_ calcd, %: C, 7.74; H, 1.95; N, 2.26; found, %: C, 7.91; H, 2.04; N, 2.38.

### 2.2. Synthesis of ***2***

The procedure was the same as for **1**, using Bi_2_O_3_ (93 mg, 0.2 mmol) instead of Sb_2_O_3_. Yield 82%. For C_12_H_36_N_3_Bi_2_I_11_ calcd, %: C, 7.07; H, 1.78, N, 2.06; found, %: C, 7.16, H, 1.89; N, 2.20.

### 2.3. Synthesis of ***3***

29 mg (0.1 mmol) of Sb_2_O_3_, 67 mg (0.3 mmol) of 1-MePyI and 25 mg (0.1 mmol) of I_2_ were dissolved in 3.5 mL of HI (70 °C, stirring, 30 min). Resulting solution was slowly cooled to r.t. Partial evaporation of the solvent results in formation of dark crystals. Yield 64%. For C_18_H_24_N_3_Sb_2_I_11_ calcd, %: C, 11.25; H, 1.26; N, 2.19; found, %: C, 11.31; H, 1.32; N, 2.29.

### 2.4. Synthesis of ***4***

The procedure was the same as for **3**, using Bi_2_O_3_ (47 mg, 0.1 mmol) instead of Sb_2_O_3_. Yield 79%. For C_18_H_24_N_3_Bi_2_I_11_ calcd, %: C, 10.30; H, 1.15, N, 2.00; found, %: C, 10.41, H, 1.27; N, 2.14.

### 2.5. X-ray Diffractometry

Crystallographic data and refinement details for **1**–**4** are given in Table S1 (Supplementary Materials). For **1**, **2** and **4**, the data were collected on a Bruker D8 Venture diffractometer with a CMOS PHOTON III detector and IµS 3.0 source (Mo Kα radiation, λ = 0.71073 Å) at 150 K. The φ- and ω-scan techniques were employed. Absorption correction was applied by SADABS (Bruker Apex3 software suite: Apex3, SADABS-2016/2 and SAINT, version 2018.7-2; Bruker AXS Inc.: Madison, WI, USA, 2017). For **3**, the data were collected on a New Xcalibur (Agilent Technologies) diffractometer with MoK_α_ radiation (λ = 0.71073) by doing φ scans of narrow (0.5°) frames at 150 K. Absorption correction was done empirically using SCALE3 ABSPACK (CrysAlisPro, Agilent Technologies, Version 1.171.37.35 (release 13 August 2014 CrysAlis171.NET). Structures were solved by SHELXT [23] and refined by full-matrix least-squares treatment against |F|^2^ in anisotropic approximation with SHELX 2014/7 [24] in ShelXle program [25].

CCDC 2214619-2214621 contain the supplementary crystallographic data for **1**, **3** and **4** correspondingly. These data can be obtained free of charge via http://www.ccdc.cam.ac.uk/conts/retrieving.html, or from the Cambridge Crystallographic Data Centre, 12 Union Road, Cambridge CB2 1EZ, UK; fax: (+44) 1223-336-033; or e-mail: deposit@ccdc.cam.ac.uk.

### 2.6. Powder X-ray Diffractometry (PXRD),

XRD analysis of polycrystals was performed on Shimadzu XRD-7000 diffractometer (CuK-alpha radiation, Ni—filter, linear One Sight detector, 0.0143° 2θ step, 2s per step). Plotting of PXRD patterns and data treatment was performed using X’Pert Plus software (see Supplementary Materials).

### 2.7. Raman Spectroscopy, Thermogravimetric Analysis, Difuse Reflectance Spectroscopy

Details are given in Supplementary Materials.

## 3. Results and Discussion

There is a general method for preparation of polyhalogen-halometalates. There are three components: (1) metal source (oxide, oxohalide or halide), which is converted into halometalate by addition of either hydrohalic acid or other source of halide ligand, (2) halide salt of certain cation and (3) dihalogen. Depending on the precursors, synthesis can be conducted in acidic aqueous solutions or in organic solvents, such as acetonitrile. Preparation of **1**–**4** follows this paradigm as well, with no specific details (see Experimental Section). The yields can be regarded as good.

The main feature of the pairs **1**–**2** and **3**–**4** is that each of them is isostructural. In all cases, the halometalate building blocks are binuclear [Sb_2_I_9_]^3−^ or [Bi_2_I_9_]^3−^ anions consisting of two {MI_6_} octahedra connected via shared face. This type of anion is very common in structural chemistry of Group 15 halometalates [26,27,28,29,30]. The M-I_term_ and M-μ_2_-I bond lengths in **1**–**4** are given in Table 1.

In the crystal structure of **1**, the [Sb_2_I_9_]^3−^ units have orientational disorder over two closed positions with 0.97/0.03 occupancies. All TMA^+^ cations are disordered over two different orientations with different occupancies. In the case of complex **2**, the quality of crystalline material was too low to extract enough data for well-solved crystal structure. Complex **2** has practically the same unit cell parameters as complex **1**. Therefore, the use of structural atomic coordinates from **1** in the refinement of 2 gave the structural model with R c.a. 12%.

The main structural feature of **1**–**4** is the presence of diiodine units which, as follows from analysis of interatomic distances (<3.98 Å, which is the sum of Bondi’s van der Waals radii [31]), likely interact with terminal iodide ligands of [M_2_I_9_]^3−^ anions. The geometric parameters of M-I···I and I-I···I fragments are given in Table 1.

**Figure 1:**
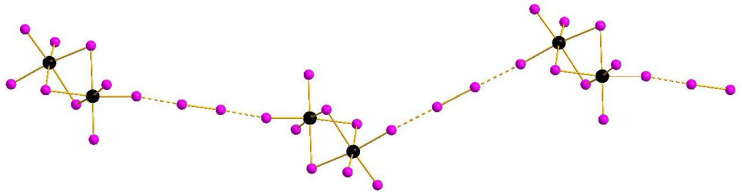
{[M_2_I_9_](I_2_)} supramolecular chains in the structures of **1** and **2**. Here and below: Sb or Bi black, I purple, non-covalent interactions dashed.

**Figure 2:**
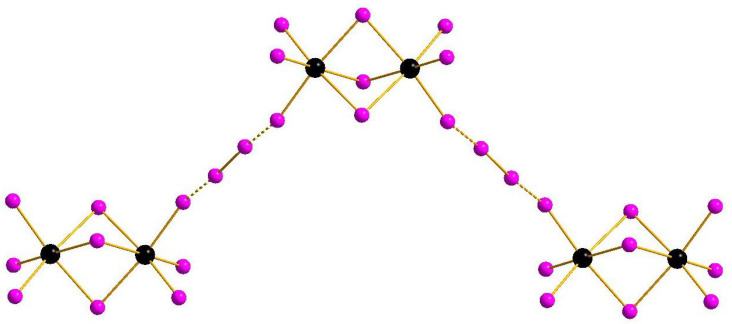
{[M_2_I_9_](I_2_)} supramolecular chains in the structures of **3** and **4**.

Complexes of general formula {[M_2_X_9_](X_2_)} were known earlier for M = Sb [32] and Bi [33], demonstrating isomerism due to diverse systems of halogen···halogen interactions. This feature appears also in the pairs **1**–**2** and **3**–**4**: mutual orientation of [M_2_I_9_]^3−^ and I_2_ building blocks vary, resulting in assembly of 1D supramolecular chains of different shape (Figure 1 and Figure 2). The crystal packings for the pairs **1**–**2** and **3**–**4** are given in Supplementary Materials.

Powder X-ray diffractometry data indicate that **1**–**4** are precipitated as pure single phases (see Supplementary Materials, Figures S1–S4). This fact makes possible in-depth investigation of physical properties of these compounds.

**Figure 3:**
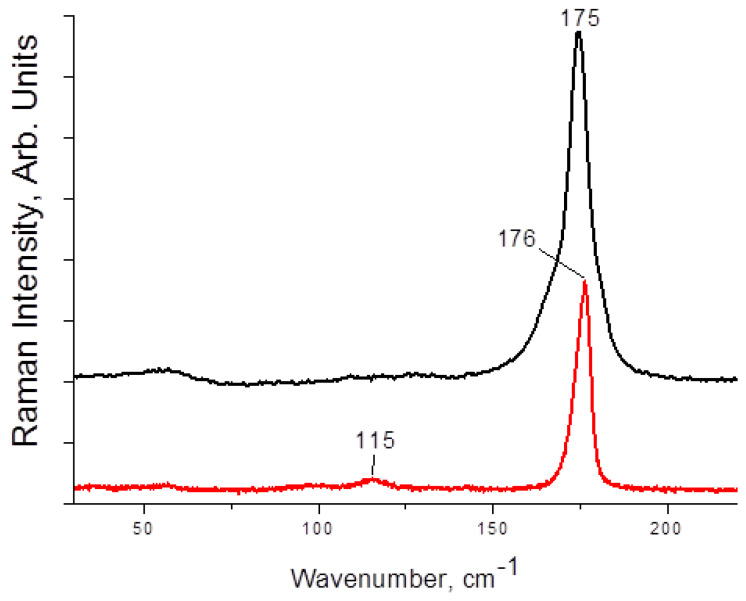
Raman spectrum of **1** (black) and **2** (red).

**Figure 4:**
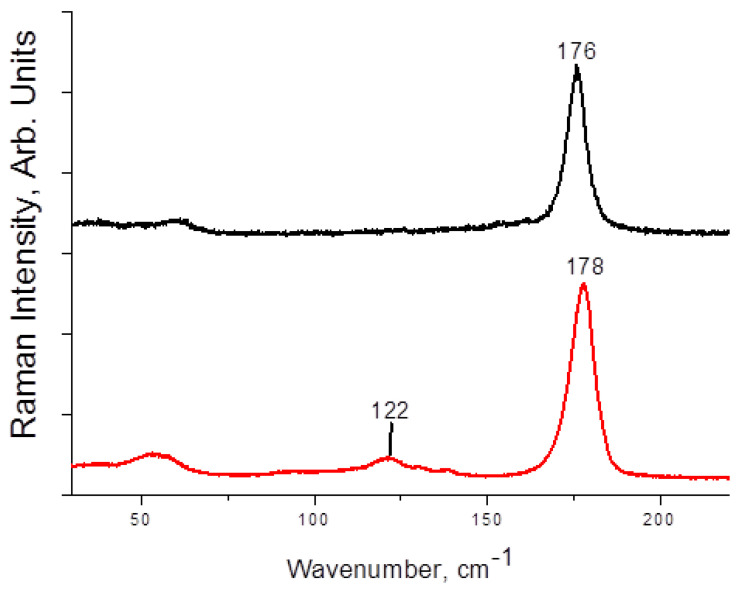
Raman spectrum of **3** (black) and **4** (red).

Raman spectra of **1**–**4** are presented on Figure 3 and Figure 4. The main and most characteristic spectral feature appears at 175–178 cm^−1^; it corresponds to the diiodine fragment. Interestingly, this band is not metal-sensitive (the differences are minor), and it is not strongly affected by the variations in I_term_···I_I2_ distances (Table 1). This fact agrees well with the Raman data obtained by us earlier for other diiodine-iodobismuthates(III): the range of shifts for incorporated I_2_ is rather narrow (170–175 cm^−1^ [22,34]). The bands at <150 cm^−1^ are very weak (also similar to the earlier results [22]); those correspond to different modes of {MI_6_} octahedra.

Overall, these observations confirm that the use of Raman spectroscopy can shed light on the nature of polyhalide units incorporated into the polyhalogen-halometalate framework or just indicate their presence (in express analysis of the outcomes of such syntheses).

**Figure 5:**
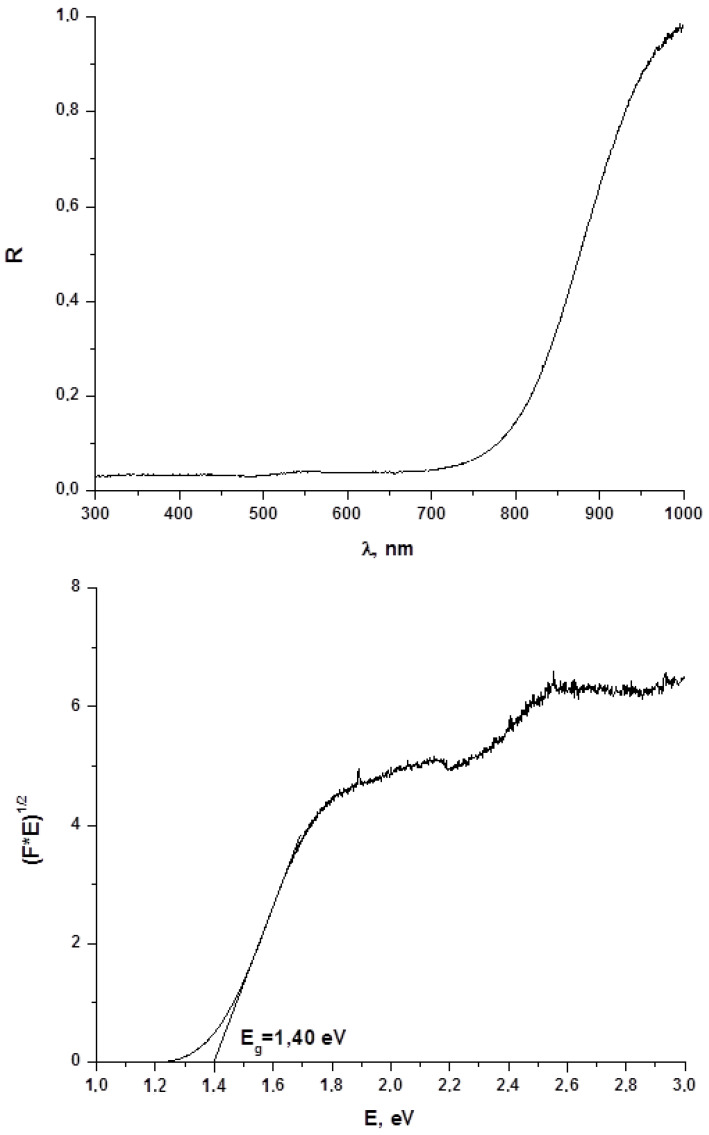
Diffuse reflectance spectrum and determination of optical band gap for **1**.

Diffuse reflectance spectroscopy is a powerful method for evaluation of optical band gap values of both bulk phases and thin films, and this parameter is crucial for estimation of whether obtained materials are promising for further use in photovoltaics. On this reason, we characterized **1**–**4** by this technique. Results are demonstrated on Figure 5 (for **1**) and in Supplementary Materials (for **2**–**4**, respectively; Figures S5–S10). As follows from these data, the E_g_ values for **1**–**4** are 1.40, 1.45, 1.42 and 1.49 eV, respectively, being lower than one for BiI_3_ (≈ 1.7 eV [16]). On one hand, these data agree well with those obtained for other representatives of this class of compounds [22], on another, those clearly demonstrate that optical properties of **1**–**4** make these compounds suitable for further photovoltaic tests.

Another very important parameter is thermal stability of halometalate hybrids. For its estimation, we used thermogravimetric analysis (TGA). As follows from these data (see Supplementary Materials, Figures S11–S14), antimony- and bismuth-containing complexes demonstrate different behavior. For Sb(III) derivatives, the loss of incorporated I_2_ is gradual, and it begins almost simultaneously with the start of heating. For iodobismuthates(III), this stage of thermal decomposition has a narrower temperature range (Figures S12 and S14), and it can be stated that **2** and **4** are stable at least up to 80 and 110 °C, respectively. These facts allow us suggesting that **4** is the most suitable candidate for possible photovoltaic studies.

## 4. Conclusions

We expanded the series of diiodine-halometalates of Group 15 elements (antimony and bismuth) by two isostructural pairs belonging to two different isomers of {[M_2_I_9_](I_2_)}^3n−^ type. Although all prepared complexes feature optical band gaps favorable for photovoltaic applications, only one compound (**4**) demonstrated higher thermal stability making such tests practically reasonable. We believe, however, that the studies of polyiodo-iodometalates in terms of photovoltaics will continue within the upcoming years.

## Figures and Tables

**Table 1:** Selected geometric parameters in **1**–**4**.

	1	3	4
M-I_term_	2.820–2.950	2.830–2.935	2.909–2.998
M-μ_2_-I	3.088–3.329	3.154–3.281	3.207–3.306
I_term_···I_I2_, Å	3.259	3.415	3.410
I-I (in I_2_), Å	2.743–2.746	2.731	2.725
M-I_term_-I_i2_,°	165.67–168.67	137.79	136.63
I_term_-I_i2_-I_i2_,°	175.48–177.55	170.20	168.26

## Data Availability

Not applicable.

## Supplementary Materials

The following supporting information can be downloaded at: https://www.mdpi.com/article/10.3390/molecules27238487/s1, XRD, PXRD and TGA data, UV-Vis spectra, Raman spectroscopy details.
