# Supplementary material for: One-Dimensional Iodoantimonate(III) and Iodobismuthate(III) Supramolecular Hybrids with Diiodine: Structural Features, Stability and Optical Properties
Source: Molecules. 2022 Dec 2;27(23):8487. doi: 10.3390/molecules27238487 (PMC9735928; doi:10.3390/molecules27238487)
Supplement: Supplementary file 1 [file molecules-27-08487-s001.zip › molecules-2017438-supplementary.pdf]

One-dimensional iodoantimonate(III) and iodobismuthate(III) supramolecular hybrids with diiodine: structural features, stability and optical properties

Nikita A. Korobeynikov, Andrey N. Usoltsev, Pavel A. Abramov, Maxim N. Sokolov and Sergey A. Adonin

Table S1. SCXRD Experimental details

	**(1)**	**(3)**	**(4)**
Chemical formula	$C_{12}H_{36}I_{11}N_3Sb_2$	$C_{18}H_{24}I_{11}N_3Sb_2$	$C_{18}H_{24}Bi_2I_{11}N_3$
M_r	1861.84	1921.80	2096.26
Crystal system, space group	Triclinic, $P\bar{1}$	Orthorhombic, *Pnma*	Orthorhombic, *Pnma*
a, *b*, *c* (Å)	9.8587 (5), 9.9097 (5), 23.8150 (12)	15.8015 (4), 22.9866 (7), 11.1179 (3)	15.917 (2), 23.152 (3), 11.1402 (18)
α, β, γ (°)	92.448 (2), 91.697 (2), 118.293 (2)	90, 90, 90	90, 90, 90
V (Å^3)	2043.54 (18)	4038.27 (19)	4105.4 (11)
Z	2	4	4
μ (mm^{-1})	9.64	9.76	16.85
Crystal size (mm)	0.55 × 0.06 × 0.01	0.25 × 0.25 × 0.02	0.13 × 0.05 × 0.03
Diffractometer	Bruker D8 Venture diffractometer	New Xcalibur, AtlasS2	Bruker D8 Venture diffractometer
Absorption correction	Multi-scan *SADABS* 2016/2: Krause, L., Herbst-Irmer, R., Sheldrick G.M. & Stalke D., J. Appl. Cryst. 48 (2015) 3-10	Multi-scan *CrysAlis PRO* 1.171.38.41 (Rigaku Oxford Diffraction, 2015) Empirical absorption correction using spherical harmonics, implemented in SCALE3 ABSPACK scaling algorithm.	Multi-scan *SADABS* 2016/2: Krause, L., Herbst-Irmer, R., Sheldrick G.M. & Stalke D., J. Appl. Cryst. 48 (2015) 3-10
T_{min}, T_{max}	0.577, 0.745	0.876, 1.000	0.455, 0.746
No. of measured, independent and observed [$I > 2\sigma(I)$] reflections	29719, 8224, 7207	13094, 4695, 4049	27563, 6955, 5784
R_{int}	0.040	0.026	0.049
θ values (°)	θ_{max} = 26.4, θ_{min} = 1.7	θ_{max} = 28.9, θ_{min} = 2.0	θ_{max} = 31.5, θ_{min} = 1.8
$(\sin \theta/\lambda)_{max}$ (Å^{-1})	0.625	0.680	0.735
Range of *h*, *k*, *l*	$-11 \le h \le 12$, $-12 \le k \le 11$, $-29 \le l \le 29$	$-15 \le h \le 21$ $-22 \le k \le 31$, $-14 \le l \le 14$	$-22 \le h \le 22$, $-22 \le k \le 34$, $-16 \le l \le 15$
$R[F^2 > 2\sigma(F^2)]$, $wR(F^2)$, S	0.051, 0.125, 1.09	0.025, 0.042, 1.07	0.030, 0.060, 1.06
No. of reflections, parameters, restraints	8224, 278, 2	4695, 165, 0	6955, 165, 0
H-atom treatment	H-atom parameters constrained	H atoms treated by a mixture of independent and constrained refinement	H atoms treated by a mixture of independent and constrained refinement
Weighting scheme	$w = 1/[\sigma^2(F_o^2) + 78.1896P]$ where $P = (F_o^2 + 2F_c^2)/3$	$w = 1/[\sigma^2(F_o^2) + (0.0118P)^2]$ where $P = (F_o^2 + 2F_c^2)/3$	$w = 1/[\sigma^2(F_o^2) + (0.014P)^2 + 0.0095P]$ where $P = (F_o^2 + 2F_c^2)/3$
$\Delta\rho_{max}$, $\Delta\rho_{min}$ (e Å^{-3})	1.67, -1.27	0.67, -1.11	1.01, -1.78

\

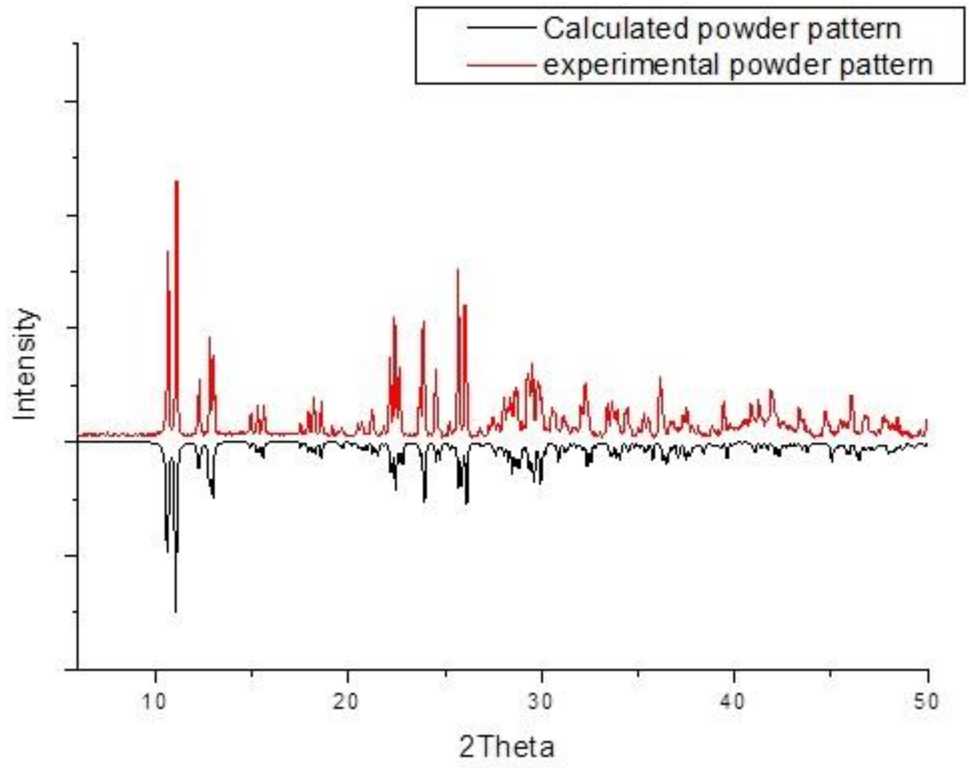

Figure S1. PXRD data for **1**

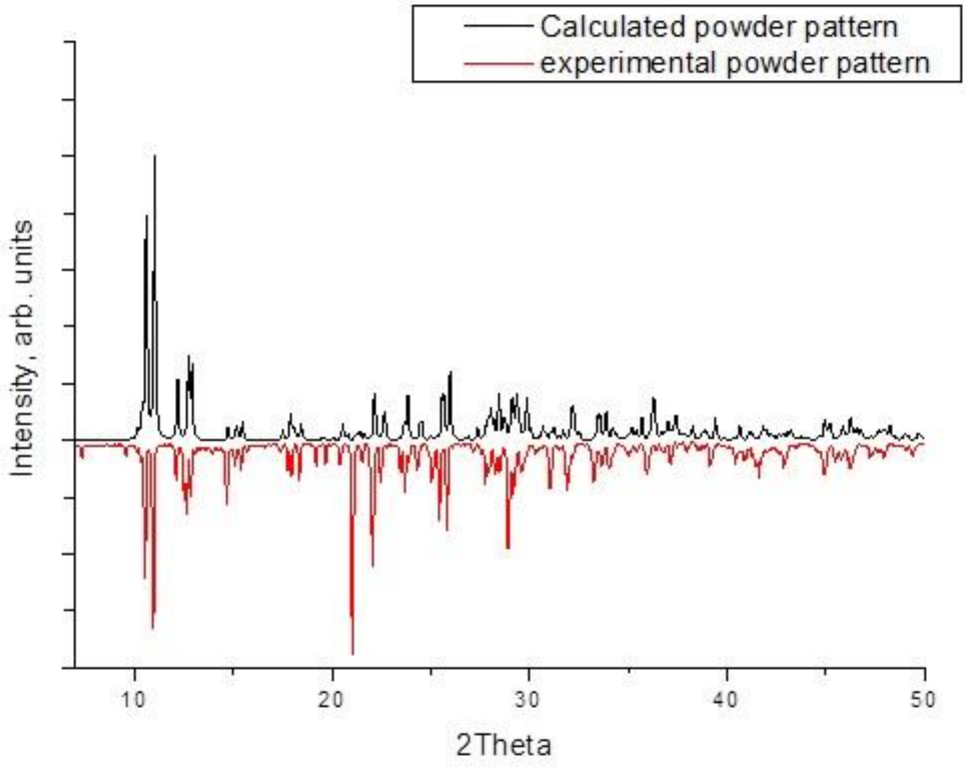

Figure S2. PXRD data for **2**

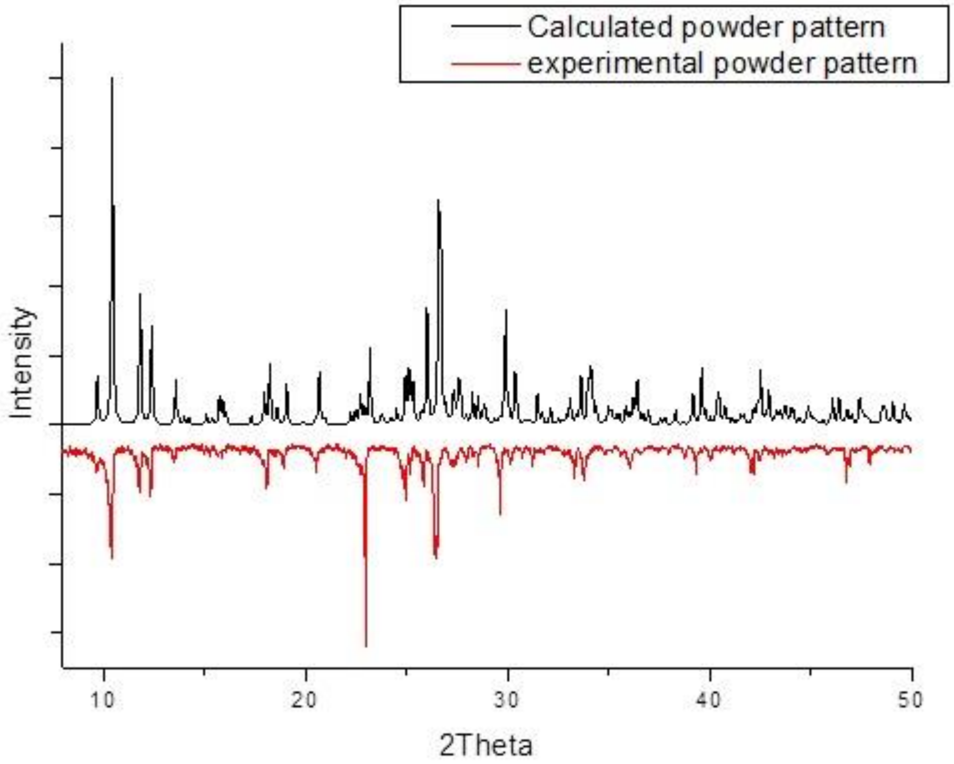

Figure S3. PXRD data for **3**

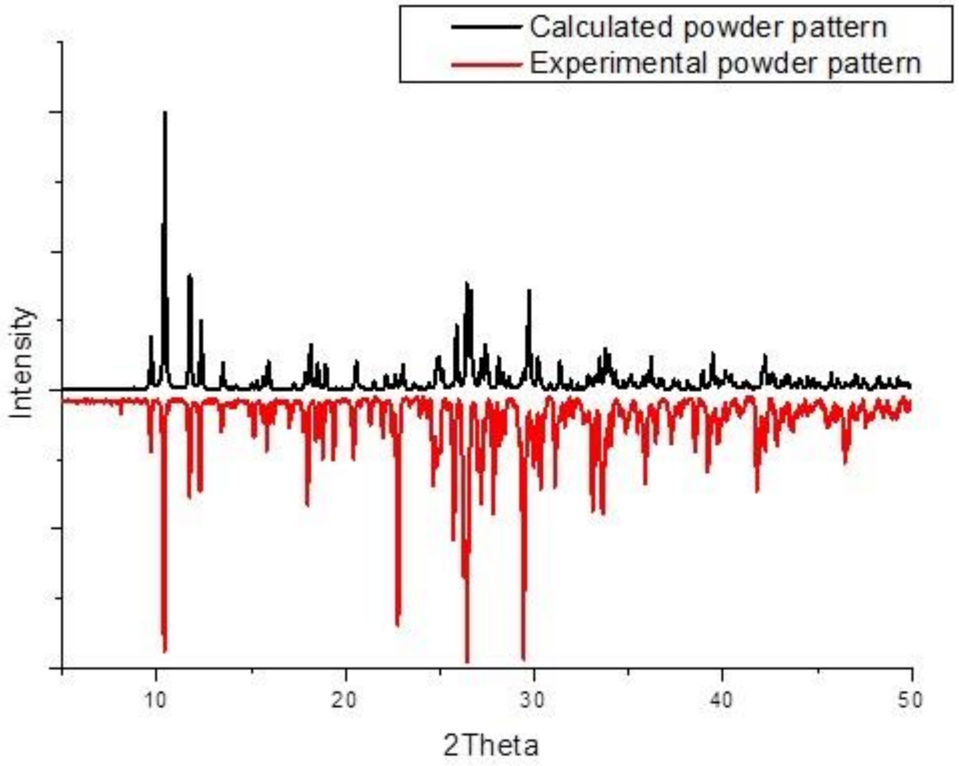

Figure S4. PXRD data for **4**

Raman spectroscopy

Raman spectra were collected using a LabRAM HR Evolution (Horiba) spectrometer with the excitation by the 633 nm line of the He-Ne laser. The spectra at room temperatures were

obtained in the backscattering geometry with a Raman microscope. The laser beam was focused to a diameter of 2 micrometers using a LMPlan FL 50x/0.50 Olympus objective. The spectral resolution was 0.7 cm-1. The laser power on the sample surface was about 0.03 mW.

Diffuse reflectance spectroscopy

Diffuse reflectance spectra were measured on an setup which consists of a Kolibri-2 spectrometer (VMK Optoelektronica, Russia), fiber optic cable QR-400-7 (Ocean Optics, USA), and deuterium–tungsten lamp AvaLight-DHS (Avantes, Netherlands). The reference of 100% reflectance was $BaSO_4$ powder. The spectra were recorded five times in the wavelength interval of 300-1000 nm and then averaged to reduce the random error.

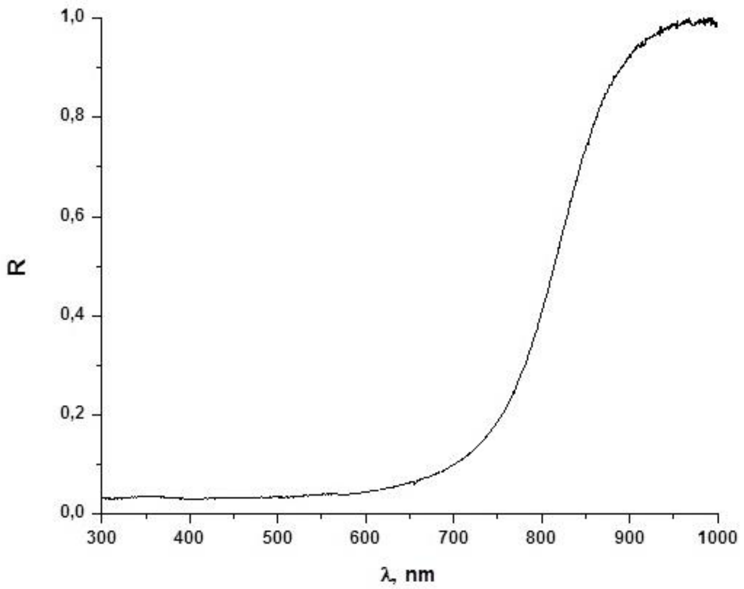

Figure S5. Diffuse reflectance spectrum for **2**

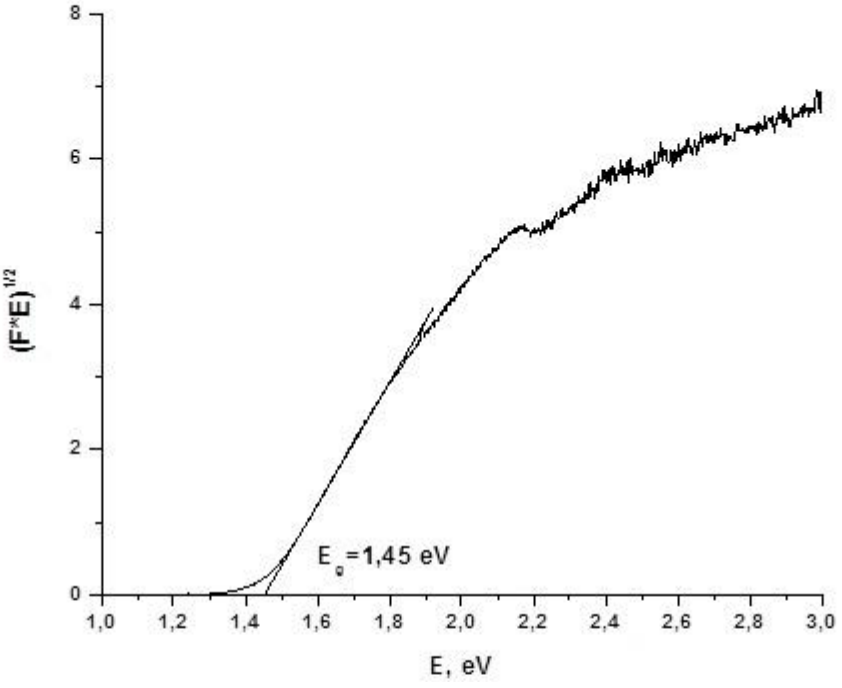

Figure S6. Band gap determination for **2**

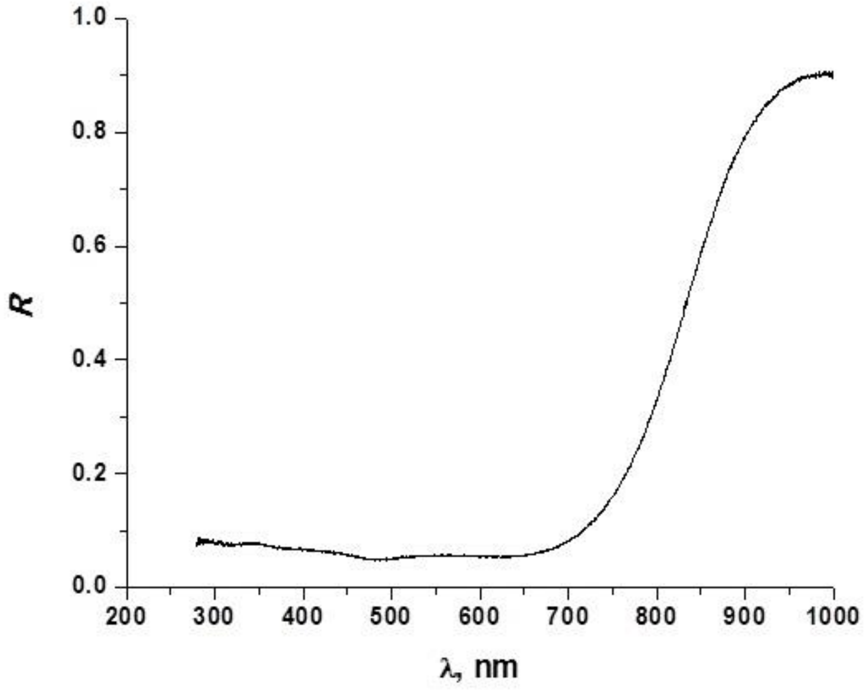

Figure S7. Diffuse reflectance spectrum for **3**

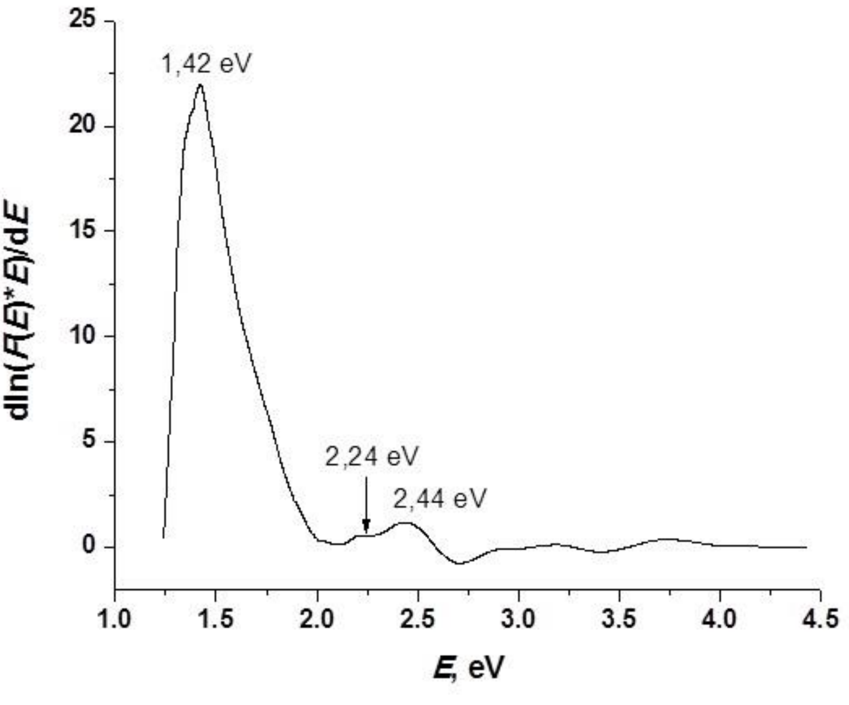

Figure S8. Band gap determination for **3**

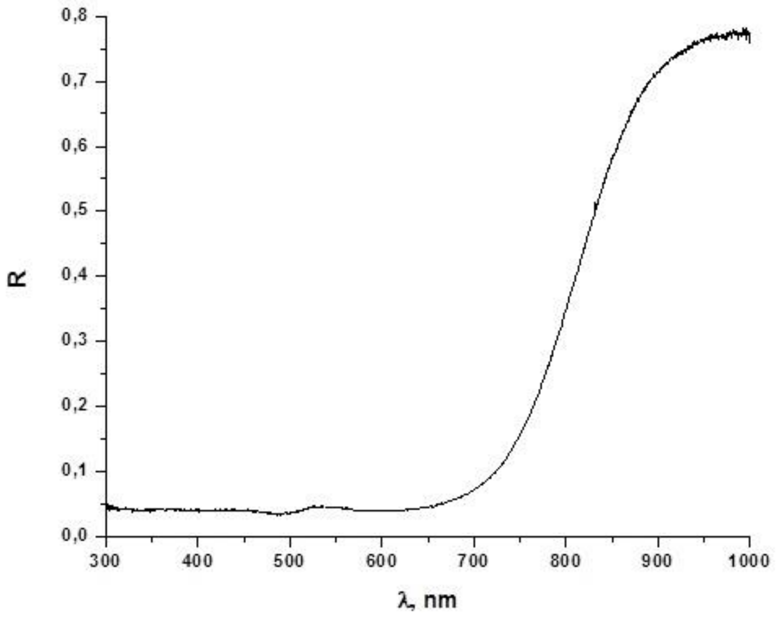

Figure S9. Diffuse reflectance spectrum for **4**

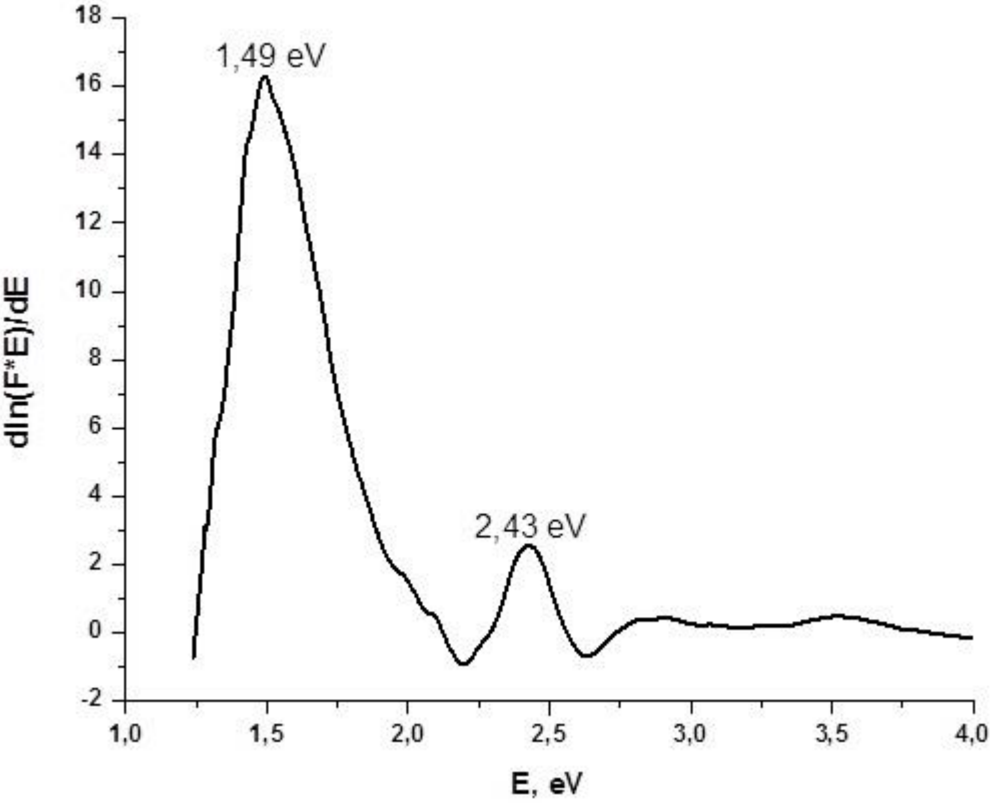

Figure S10. Band gap determination for **4**

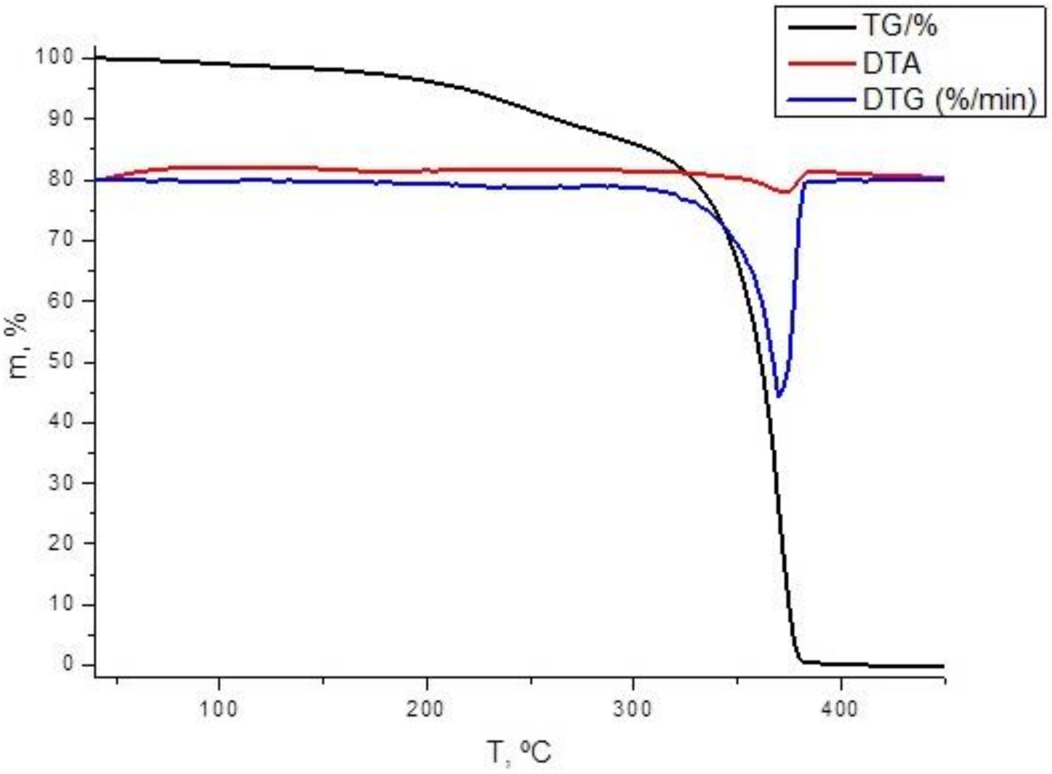

Figure S11. TG, DTA and DTG data for **1**

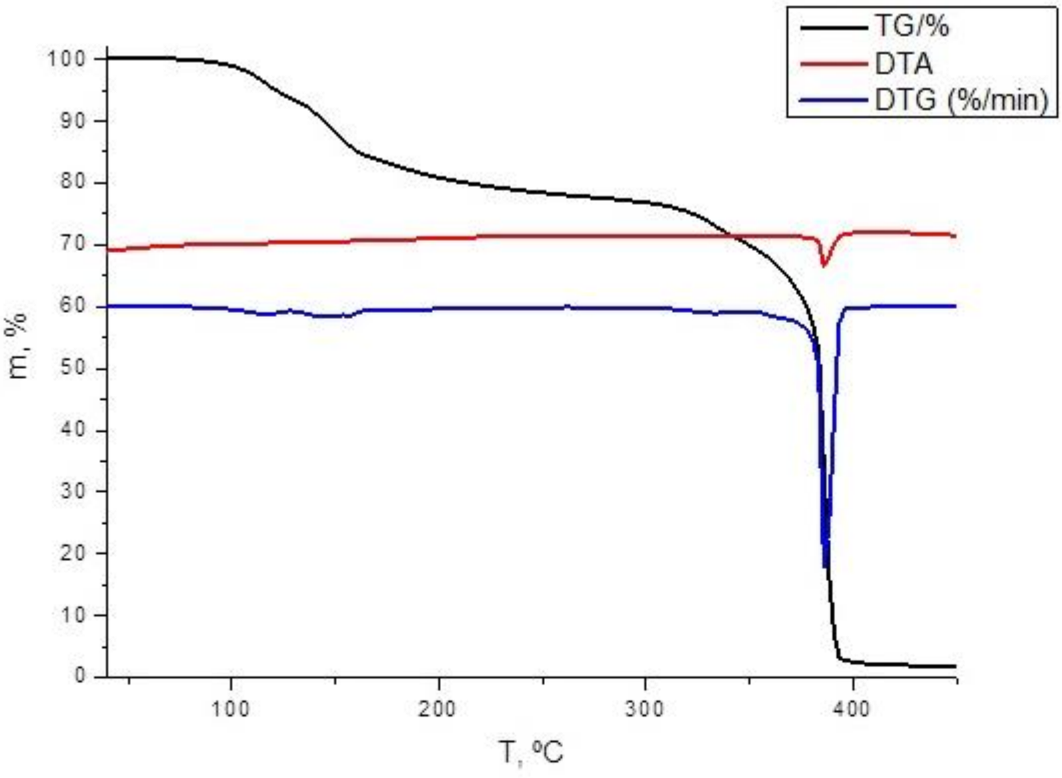

Figure S12. TG, DTA and DTG data for **2**

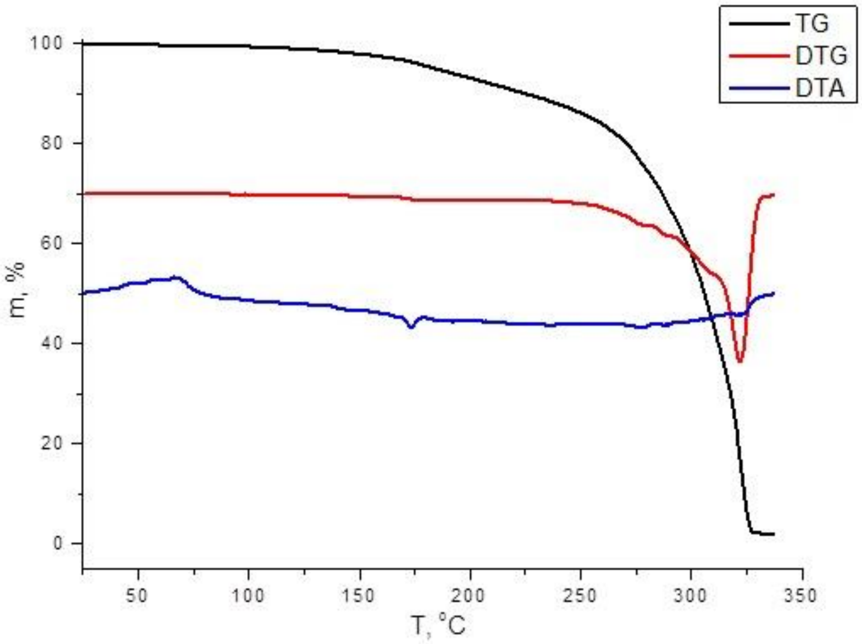

Figure S13. TG, DTA and DTG data for **3**

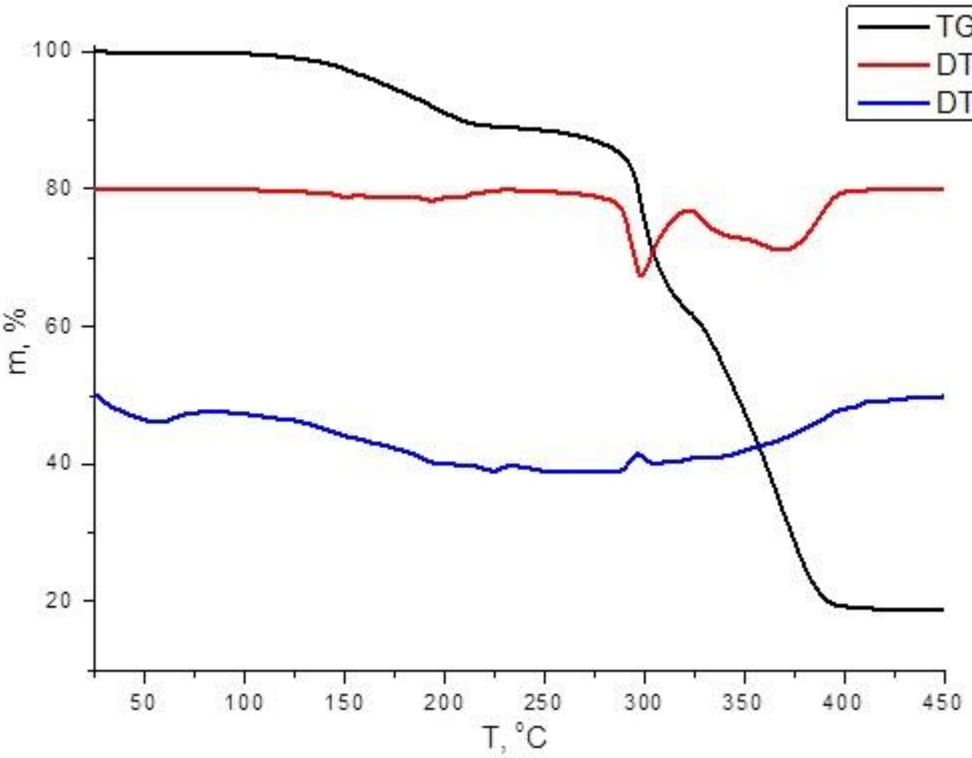

Figure S14. TG, DTA and DTG data for **4**
